# Human and Machine Intelligence Together Drive Drug Repurposing in Rare Diseases

**DOI:** 10.3389/fgene.2021.707836

**Published:** 2021-07-28

**Authors:** Anup P. Challa, Nicole M. Zaleski, Rebecca N. Jerome, Robert R. Lavieri, Jana K. Shirey-Rice, April Barnado, Christopher J. Lindsell, David M. Aronoff, Leslie J. Crofford, Raymond C. Harris, T. Alp Ikizler, Ingrid A. Mayer, Kenneth J. Holroyd, Jill M. Pulley

**Affiliations:** ^1^Vanderbilt Institute for Clinical and Translational Research, Vanderbilt University Medical Center, Nashville, TN, United States; ^2^Department of Chemical and Biomolecular Engineering, Vanderbilt University, Nashville, TN, United States; ^3^Division of Rheumatology and Immunology, Department of Medicine, Vanderbilt Medical Center, Nashville, TN, United States; ^4^Department of Biostatistics, Vanderbilt University School of Medicine, Nashville, TN, United States; ^5^Division of Infectious Diseases, Department of Medicine, Vanderbilt University Medical Center, Nashville, TN, United States; ^6^Department of Obstetrics and Gynecology, Vanderbilt University Medical Center, Nashville, TN, United States; ^7^Department of Pathology, Microbiology and Immunology, Vanderbilt University Medical Center, Nashville, TN, United States; ^8^Division of Nephrology, Department of Medicine, Vanderbilt University Medical Center, Nashville, TN, United States; ^9^Division of Hematology/Oncology, Vanderbilt-Ingram Cancer Center, Vanderbilt University Medical Center, Nashville, TN, United States; ^10^Center for Technology Transfer and Commercialization, Vanderbilt University, Nashville, TN, United States

**Keywords:** drug repurposing, evidence synthesis, rare diseases, machine learning, phenome wide association studies, precision medicine

## Abstract

Repurposing is an increasingly attractive method within the field of drug development for its efficiency at identifying new therapeutic opportunities among approved drugs at greatly reduced cost and time of more traditional methods. Repurposing has generated significant interest in the realm of rare disease treatment as an innovative strategy for finding ways to manage these complex conditions. The selection of which agents should be tested in which conditions is currently informed by both human and machine discovery, yet the appropriate balance between these approaches, including the role of artificial intelligence (AI), remains a significant topic of discussion in drug discovery for rare diseases and other conditions. Our drug repurposing team at Vanderbilt University Medical Center synergizes machine learning techniques like phenome-wide association study—a powerful regression method for generating hypotheses about new indications for an approved drug—with the knowledge and creativity of scientific, legal, and clinical domain experts. While our computational approaches generate drug repurposing hits with a high probability of success in a clinical trial, human knowledge remains essential for the hypothesis creation, interpretation, “go-no go” decisions with which machines continue to struggle. Here, we reflect on our experience synergizing AI and human knowledge toward realizable patient outcomes, providing case studies from our portfolio that inform how we balance human knowledge and machine intelligence for drug repurposing in rare disease.

## Introduction

In today’s age of big healthcare data, how should artificial intelligence (AI) and human critical thinking and creativity coexist within the drug development process? The long-range effects of integrating AI tools such as machine learning (ML) into drug development remain to be determined, particularly for complex problems such as rare disease strategies. Within the burgeoning field of drug repurposing—which seeks to facilitate and accelerate drug development by discovering new use cases for existing drugs (Nosengo, 2016)—this discussion is especially timely. The oldest, most well-known cases of repurposing, such as sildenafil for erectile dysfunction (Roundtable on Translating Genomic-Based Research for Health et al., 2014), are often considered serendipity but were in reality unexpected effects astutely recognized by humans. As procedures and knowledge bases for the identification and validation of repurposing candidates continue to mature and grow (Challa et al., 2018; Xue et al., 2018; Pulley et al., 2020), the field is extremely fertile for the application of AI to identify patterns suggestive that a drug candidate is attractive for repurposing.

Repurposing of existing therapeutic agents has generated significant interest in the realm of rare diseases as an innovative strategy for finding new opportunities to manage these complex conditions (Delavan et al., 2018; Valencic et al., 2018; Scherman and Fetro, 2020; Roessler et al., 2021). As data resources that integrate longitudinal phenotype information with genetic data and patient demographics continue to expand and evolve, so does their utility for identifying new therapeutic insights for an array of rare diseases. Several studies have harnessed these data with ML to identify drug repurposing candidates, exploring connections among multi-omics data with potential therapeutic implications (Jarada et al., 2020; Chen et al., 2021). Drug discovery in rare disease has been a particularly innovative use case for this approach, allowing investigators to predict molecular etiologies for rare diseases from existing knowledge on illnesses with similar presentations. Subsequently, these predictions can inform a shortlist of repurposable therapies for such rare diseases. This is done by considering the therapeutic indices of agents indicated for those illnesses that ML identifies as having pathophysiology and symptomology similar to the rare disease of interest (Álvarez-Machancoses and Fernández-Martínez, 2019; Brasil et al., 2019; Lee et al., 2019). The power of ML in this context is in its capacity to identify patterns at scale. ML’s automation of this discovery step expedites the pace of hit discovery and expands the landscape of potential candidates translatable to future stages of the development pipeline. This approach’s efficiency is magnified when the probability of this kind of a hit is otherwise low, as it is for many rare disease therapeutic campaigns (Ekins et al., 2019).

Despite the promise, current ML architectures struggle to parse the diverse, semi-structured feature sets inherent to the available data. Thus, we propose that ML can be a supplement—but not a replacement—for the perspectives of domain experts in drug repurposing. The complementary and interconnected nature of human intelligence and AI is becoming apparent (Kim et al., 2021). We assert that in its current state ML is best leveraged in drug repurposing efforts to inform human “go/no-go” decision-making. Relying on rigid rules-based criteria typically required by ML overlooks that important information remains insufficiently codified to even be able to apply a rule.

Our drug repurposing team at Vanderbilt University Medical Center (VUMC) synergizes computational techniques like the phenome-wide association study (PheWAS) (Denny et al., 2010, 2013)—an ML method for generating hypotheses about new indications for an approved drug—with data mining from public databases (Challa et al., 2019). As we describe throughout the case studies we present in this manuscript, PheWAS inputs phenotypes encoded as administrative billing codes [e.g., the International Classification of Diseases ontology (World Health Organization, 1993; Centers for Disease Control and Prevention, 2021)] and tests the strength of associations between the presence of these codes in patients’ electronic health records (EHRs) and the incidence of single nucleotide mutations recorded for these patients within clinical genomics repositories that accompany their EHRs. The associations are formalized through a logistic classification algorithm, which provides *p*-values (correctable for multiple testing) and odds ratios to quantitatively assess the strength of each predicted mutation-disease pair (Denny et al., 2010, 2013). These data are then overlaid with the knowledge of scientific and clinical domain experts, which facilitates review of true causality, identification of drugs associated with the targets carrying implicated mutations on their encoding genes, as well as the likelihood of successfully repurposing these drugs for the diseases indicated by PheWAS. In this article, we reflect on the processes involved in our program, with a view to areas of synergy for ML approaches like PheWAS and human knowledge. We share illustrative examples from our portfolio that support our beliefs in the unique balance of human and machine intelligence required for drug repurposing in rare disease.

## Overview of the Drug Repurposing Platform at Vanderbilt University Medical Center

Our drug repurposing platform at VUMC (Pulley et al., 2017, 2018a,b; Goldstein et al., 2018; Jerome et al., 2018; Choby et al., 2019) leverages natural human genetic variation as a proxy for, and method for more accurately predicting, the physiologic effects of therapies in humans. The key resource enabling this work is BioVU, a repository of 245,000 unique, de-identified DNA samples derived from discarded blood collected during routine clinical testing (Roden et al., 2008). BioVU, combined with extensive and granular phenotype data from the EHR, serves as a centralized resource for conducting largescale disease-agnostic research on fundamental questions of how genetic variation corresponds to variations in observable attributes, like the PheWAS analyses described above. The open-source codebase enabling execution of PheWAS and replication of previously published PheWAS signals (Pulley et al., 2017; Jerome et al., 2020) and a catalog of single nucleotide polymorphism (SNP)/phenotype findings are publicly available online (Denny et al., 2013; PheWAS, 2021).

**FIGURE 1:**
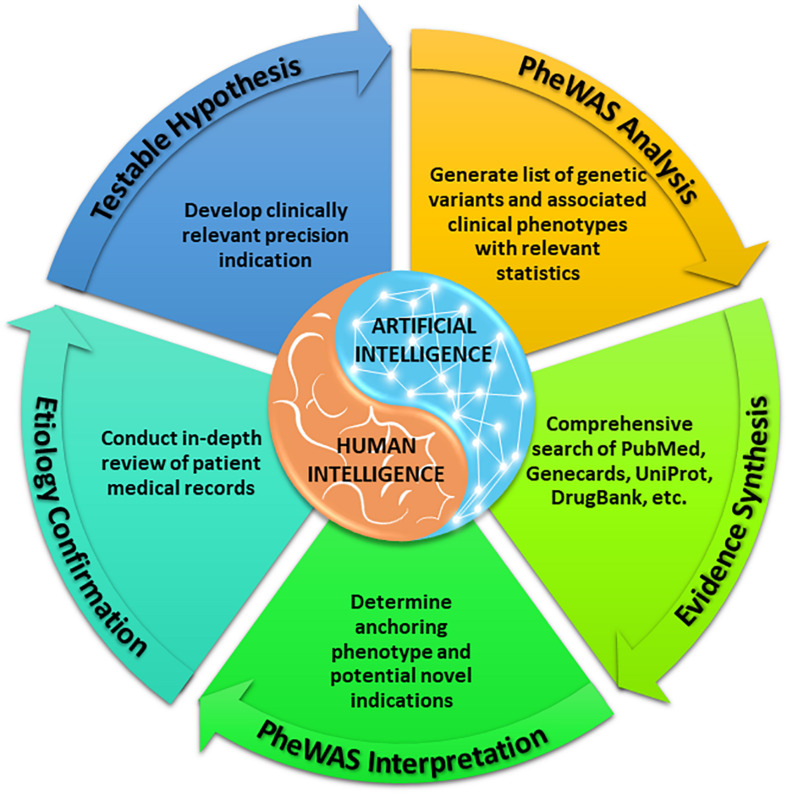
VUMC Drug Repurposing Platform methods for establishing precision indications.

With a sufficiently large dataset, SNPs with low minor allele frequencies can allow for assessment of therapeutic targets among rare diseases, given the size and breadth of data yielded by large, diverse healthcare centers. As above, our methods (Figure 1) identify variants in drug target genes (Wishart et al., 2018) and then execute PheWAS (Denny et al., 2010) to find the diseases associated with these SNPs. When a known indication of a drug results from this analysis (e.g., when a SNP that is known to lower gastric acid secretion has a statistically significant association with reduced risk of heartburn), we can reasonably conclude that the SNP recapitulates the effects of a comparative drug (e.g., a proton pump inhibitor). Building upon these validation signals, we assess associations between the SNP and potential novel indications for the associated drug, as represented in phenotypes yielded by PheWAS. Our method does not solely rely on the classifier; it also requires the integration of conceptual knowledge on the clinical manifestations of human disease and markers of disease pathophysiology. To facilitate this conceptual synthesis of existing knowledge, we have developed a plausibility assessment approach with extensive evidence review using comprehensive searches of a wide range of information science resources. These methods are applied to make selections for clinical development. Using this procedure, we have established a suite of 13 drug-indication pairings, and we have generated confirmatory data for five (out of six) programs to date. Table 1 provides several illustrative examples from our program’s slate of projects.

**TABLE 1:** Example projects, Vanderbilt University Medical Center Drug Repurposing Platform.

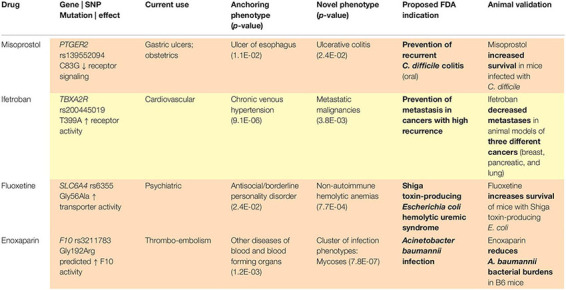

*Bold text highlights key phenotypes.*

Each project in our repurposing pipeline represents a precise disease or, when the disease is not a classical rare disease, a rare disease endotype, defined by a discrete pathophysiologic mechanism and cluster of characteristics that is revealed by review and synthesis of narrative descriptions of disease presentations (e.g., as detailed in clinical notes) within candidate EHRs. It is well known that the presentations of rare diseases are often so complex or opaque that even skilled clinicians require extensive workups and multidisciplinary consults to unearth a sufficiently discriminatory differential. ML’s goal of pattern recognition could allow for more efficient identification of these rare disease differential diagnoses, but to make accurate predictions of which drugs might work for which rare disease, the ML algorithms require knowledge of presence, absence, and corresponding significance of features to facilitate accurate rule-in/rule-out modeling. We propose that these methods do not yet have the sophistication to mirror or replace the expert domain knowledge applicable to finding new rare disease indications for existing drugs, as we have found in our repurposing experiences.

## Limitations of the Utility of AI Based on Our Drug Repurposing Experiences

Researchers are developing approaches to systematically support and expand repurposing efforts, some of which use ML technologies (Chen et al., 2021; Gupta et al., 2021; Issa et al., 2021). Promising techniques include quantitative structure-activity relationship modeling (Ekins et al., 2019; Challa et al., 2020), *in silico* docking experiments on druggable targets, virtual high-throughput screening, adverse event matching, and applying advanced statistical approaches to big clinical, genomic, pathway, and gene regulation data to discover new relationships within this information toward personalized medicine (Challener, 2018; Le et al., 2019, 2020). Despite the advantages of an AI approach like unsupervised ML to discover previously unrecognized patterns, as well as the large extent to which AI allows for pliable model development, we have found several challenges hindering the potential of AI to be fully realized in the context of our platform. While we believe that PheWAS offers an advance over other functionally similar ML approaches in its ability to work robustly across the healthcare data of diverse enterprises (Hermann et al., 2021; Salvatore et al., 2021; Schneider et al., 2021) and its empowerment of holistic, high-throughput discovery through minimal model pre-conditioning, its results require manual interpretation.

A key first step in our hypothesis generation workflow is identifying, *via* PheWAS, SNPs within a drug target’s genes that replicate known therapeutic information about the drug (i.e., known indications or side effects of the drug). Given that the indications or side effects on a drug’s label may not directly correlate to patient experiences in the real world, identification of the “controls” that underlie our models requires leveraging deep conceptual knowledge from the literature and from clinical collaborators. For example, we used “aphasia” (odds ratio = 5.05, *p* = 0.007) as an anchoring phenotype in analyzing PheWAS results for *GRIN2A*, which encodes the protein target of memantine. Memantine is approved for treatment of Alzheimer’s disease but has limited efficacy for that indication as a standalone therapy. Literature evidence indicates stronger therapeutic efficacy for aphasia (Berthier et al., 2011), which is among the top associations in our PheWAS dataset and is also a feature of neuropsychiatric lupus, which we further identified as a pathogenicity of SNPs linked to *GRIN2A.* Cognitive dysfunction in patients with systemic lupus erythematosus patients then became our repurposed indication, which we are currently interrogating through clinical testing (NCT03527472). If we had only relied on ML to construct our model, would a rules-based classifier have recognized a theme of aphasia, or the “rare” subtype of lupus, when these diseases are written in clinical narratives without formal diagnosis?

Another example of ways in which manual patient chart reviews can contribute to our process involves our use of electrolyte imbalance as the anchoring phenotype for *TACR1*, which encodes the protein target of aprepitant. Aprepitant is approved for chemotherapy-induced nausea and vomiting; review of patient charts confirmed that vomiting explained our controlling set of electrolyte imbalance PheWAS results in patients with *TACR1* mutations. Could ML stably and efficiently integrate such relationship-driven disease classification knowledge into a larger inference model, in a way that would prevent calling this true-positive result a false-negative?

Establishing parity between a drug’s mechanism of action and SNP directionality enables us to confirm known indications, identify potentially novel indications, and pair drugs and SNPs with inference regarding mode of action for a SNP effect (e.g., inhibition, activation, agonism, or antagonism). However, SNP function, especially for rare variants, is often not known. Because ML model performance is poor in the presence of data missingness, automating this critical component of our workflow is not currently feasible. Our anchoring work thus plays a key role in allowing us to infer SNP function in the absence of previous evaluation of a variant’s effect. Lastly, depending on the database or the literature source, SNP directionality is often inverted (i.e., which allele is considered “minor” or more rare as well as risk causing, which varies by population), and therefore can be misinterpreted in the exact opposite way it should be. Such catastrophic failure is unlikely to be identified by most ML frameworks.

Given that PheWAS results may contain false positives (i.e., statistically significant *p*-values that will not help the drug development process) and false negatives (i.e., weaker *p*-values that are truly meaningful to the process), automating the evaluation of PheWAS results based on *p*-values is unlikely to reliably identify the strongest repurposing indications, not to mention the multitude of cautionary tales and even the stance of the American Statistical Association against reliance on *p*-values for decision making. One example of this problem is illustrated by our program to repurpose misoprostol for prevention of *Clostridioides difficile* recurrence, which has validating preclinical data (Zackular et al., 2019) and is currently in clinical testing (NCT03617172). Rather than arriving at our precision indication directly from a single PheWAS association, we worked from a cluster of individually weaker, but strongly related, associations with *p*-values that would not individually pass corrections for multiple testing. Combining data from across multiple analyses with awareness of diagnostic code overlap represents an area in need of methodological innovation (Strayer et al., 2020).

Phenome-wide association study relies heavily on diagnostic codes. Diagnostic codes, and thus their corresponding phenotypes, are not always self-explanatory, may vary in usage patterns based on local practice, may require assessment dependent on other ontologies, and often warrant consultation with clinicians for interpretation. For example, our program to repurpose ifetroban for prevention of metastases in multiple cancer types originated with a cluster of significant associations with several “secondary cancers.” Manual review of patient charts revealed that secondary cancer codes were being used to signify metastatic spread originating from various primary tumor sites, which was essential in developing our repurposing indication; this program also has validating preclinical data (Werfel et al., 2020) and is currently in clinical testing (NCT03694249). If we had relied only on ML to construct our indication, would a rules-based classifier have recognized this rare sub-population who appear predisposed to metastases, when these diseases are coded as “secondary cancers?”

An additional shortcoming of diagnostic code-based clinical datasets is that a single disease code may represent several disease endotypes with vastly different etiologies. This is the case for our chronic fatigue syndrome (CFS) repurposing program, in which our treatment strategy would correct a disruption in norepinephrine transport. This hypothesis was derived from a PheWAS signal in the gene that encodes the norepinephrine transporter, and data from our completed biomarker study (NCT03029377) that suggest that this disruption is present in a subset of CFS patients. If using automated approaches for hypothesis generation or evidence synthesis, it would be difficult to arrive at a repurposing indication that falls under an “umbrella disease” and delineate which evidence is relevant for the specific subset of the disease from both mechanism and symptomology perspectives.

Our evidence synthesis workflow relies on manual review of publicly available gene expression data. The data can be misleading and lack specificity regarding how expression may be altered in disease states, differ based on population demographics, and vary in organ systems or by gene subunit. In addition, some proteins have tissue-or cell-specific effects that can change from positive to negative by tissue or cell, whereas others do not. This complexity of directionality and context is difficult to assess and integrate into a scalable and coherent repurposing hypothesis without significant human scientific expert interpretation. Perhaps ML methods can be trained for this purpose, but the scope of deducing such relational knowledge at industrial scale currently precludes the automation of this step in the drug discovery workflow.

Several other issues hinder automation of literature review. For example, biologic assay specificity, in terms of measurement and whether protein subunits can be detected, is sometimes not adequately described in papers; in several repurposing projects, limitations in available descriptions of enzyme-linked immunosorbent assay (ELISA) methods have required that we directly consult with ELISA vendors to assess results and relevance, in some instances uncovering notable flaws in the published literature related to human disease-related inferences. The relevance of published animal model data may also vary based on homology against a human comparator which itself varies based on the specific disease being studied. Given that some published literature contains weak study designs and/or incoherent findings, even drugs that proclaim a mechanism of effect may be found erroneous in subsequent investigations. In such situations, the inaccurate mechanism remains in the published literature and the chance of excluding it using automated methods is currently low (McEwen et al., 2010). Furthermore, a wealth of data relevant for drug repurposing is only minimally published. Additionally, there can be serious data quality issues where publicly available databases just contain completely incorrect information. A previous version of DrugBank listed ACE2 as a target of moexipril, a target of interest in COVID-19; however, when we manually reviewed the references supporting this claim none of them contained any direct evidence that moexipril had any impact on ACE2; this has since been corrected. Finally, much drug development data are proprietary and can only be gleaned by talking directly with members of original clinical development teams; such information includes the full dose range clinically tested and comprehensive understanding of biological effects and safety profiles observed over that range. Because of this, some of the richest repurposing data are unstructured.

## Discussion

Our experience is that ML models are currently unable to handle the complexity inherent to key repurposing information sources. While advances in natural language processing have been helpful in extracting unstructured and semi structured data, the complexity of data structures and sources used in the repurposing pipeline do not lend themselves to such straightforward solutions. Sometimes the judgment call about a given compound’s selectivity (or lack thereof) and how well suited the compound is for a new use balanced against other (known) pharmacodynamic, pharmacokinetics, and safety concerns is just not straightforward and requires critical thinking, intuition, and creativity to synthesize existing knowledge into a new hypothesis with a high probability of success and low likelihood of propagating errors that exist in the historical record. The deep utility and value of engaging humans in evaluating PheWAS results, placing them within the broader scientific context, weaving together various and disparate sources of data, and identifying opportunities for confirmatory scientific investigations should not be underestimated. While current ML and other AI techniques can substantially complement these efforts by helping to identify meaningful patterns for validation, it is premature to consider replacing our human-focused approach. Identifying those aspects of the pipeline that require human insights can identify those problems amenable to algorithmic innovation to accelerate the pace of novel drug discovery.

Repurposing of existing therapeutic agents has generated significant interest in the realm of rare disease treatment, as an innovative strategy for finding ways to manage these complex conditions. In conversations with pharmaceutical industry collaborators, we often receive questions about the optimal balance of machine and human decision-making necessary to support the pipeline. This question continues to intensify given the growing availability of largescale EHR data combined with genotyping that may be leveraged for drug development. By enabling precision phenotyping and connections with genomic variation, these large datasets present a particularly exciting opportunity for exploring repurposing opportunities for rare diseases. Given the importance of hypothesis generation and the dense, complex nature of critical pre-clinical data in drug development, human knowledge remains essential for interpretation and making “go-no go” decisions with which machines continue to struggle. Much of the technology necessary to automate the complete hypothesis generation and evidence synthesis processes is not yet ready or created, but by paying careful attention to the problems that require human insight we have highlighted those areas where methodological innovation might have greatest impact on the pace of discovery.

Helping us identify previously unrecognized patterns that we can then rationalize through systematic thinking and generational of a testable hypothesis is the significant value add of ML in our current workflow. It is not a replacement for the perspectives of domain experts; instead it is best leveraged in drug repurposing efforts when it is informed by content experts and then considered synergistically with human decision-making to pursue top repurposing leads through randomized controlled trials.

## Data Availability Statement

The original contributions presented in the study are included in the article/supplementary material, further inquiries can be directed to the corresponding author/s.

## Author Contributions

All authors made substantive contributions to conception of this perspective and/or participated in the acquisition, analysis, and interpretation of data for the work. APC, NMZ, RNJ, RRL, JS-R, IAM, KJH, and JMP participated in drafting the work. APC, NMZ, RNJ, RRL, JS-R, AB, CJL, DMA, LJC, RCH, TAI, KJH, and JMP revised the perspective critically for key content. All authors approved the manuscript and agreed to be accountable for all aspects of the work.

## Conflict of Interest

The authors declare that the research was conducted in the absence of any commercial or financial relationships that could be construed as a potential conflict of interest.

## Publisher’s Note

All claims expressed in this article are solely those of the authors and do not necessarily represent those of their affiliated organizations, or those of the publisher, the editors and the reviewers. Any product that may be evaluated in this article, or claim that may be made by its manufacturer, is not guaranteed or endorsed by the publisher.
